# The causal effects of intelligence and fluid intelligence on Parkinson’s disease: a Mendelian randomization study

**DOI:** 10.3389/fnagi.2024.1388795

**Published:** 2024-05-23

**Authors:** Cong Jing, Xiaojiao Zhong, XuLi Min, Hao Xu

**Affiliations:** ^1^Departments of Interventional Radiology, Affiliated Hospital of North Sichuan Medical College, Nanchong, Sichuan, China; ^2^Yilong County General Hospital (Ma’an Campus), Nanchong, Sichuan, China

**Keywords:** Parkinson’s disease, intelligence, fluid intelligence scores, Mendelian randomization (MR) analysis, causal relationship

## Abstract

**Background:**

Parkinson’s disease (PD) is a chronic neurodegenerative disease that affects the central nervous system, primarily the motor nervous system, and occurs most often in older adults. A large number of studies have shown that high intelligence leads to an increased risk of PD. However, whether there is a causal relationship between intelligence on PD has not yet been reported.

**Methods:**

In this study, Mendelian randomization (MR) analysis was performed with intelligence (ebi-a-GCST006250) and fluid intelligence score (ukb-b-5238) as exposure factors and PD (ieu-b-7) as an outcome, which the datasets were mined from the IEU OpenGWAS database. MR analysis was performed through 3 methods [MR Egger, weighted median, inverse variance weighted (IVW)], of which IVW was the primary method. In addition, the reliability of the results of the MR analysis was assessed via the heterogeneity test, the horizontal polytropy test, and Leave-One-Out (LOO). Finally, based on gene ontology (GO) and Kyoto Encyclopedia of Genes and Genomes (KEGG) databases, the genes corresponding to intelligence and fluid intelligence score related to SNPs were enriched for functional features and pathways.

**Results:**

The results of MR analysis suggested that elevated intelligence indicators can increase the risk of PD [*p* = 0.015, Odd Ratio (OR) = 1.316]. Meanwhile, fluid intelligence score was causally associated with the PD (*p* = 0.035), which was a risk factor (OR = 1.142). The reliability of the results of MR analysis was demonstrated by sensitivity analysis. Finally, the results of GO enrichment analysis for 87 genes corresponding to intelligence related SNPs mainly included regulation of synapse organization, developmental cell growth, etc. These genes were enriched in the synaptic vessel cycle, polycomb expressive complex in KEGG. Similarly, 44 genes corresponding to SNPs associated with fluid intelligence score were used for enrichment analysis. Based on the GO database, these genes were mainly enriched in regulation of developmental growth, negative regulation of neuron projection development, etc. In KEGG, 44 genes corresponding to SNPs associated with fluid intelligence score were enriched in signaling pathways including Alzheimer’s disease, the cellular senescence, etc.

**Conclusion:**

The causal relationships between intelligence and fluid intelligence scores, and PD were demonstrated through MR analysis, providing an important reference and evidence for the study of PD.

## 1 Introduction

Parkinson’s disease (PD) is a chronic, progressive neurodegenerative disease characterized by motor and non-motor features (Tolosa et al., 2006). PD is the second most common neurodegenerative disease affecting the health of middle-aged and elderly people, second only to Alzheimer’s disease (Paul et al., 2018). The main pathological feature of PD is the gradual loss of dopaminergic neurons in the substantia nigra of the midbrain, which leads to a lack of dopamine in the striatum, causing pathophysiological changes in the downstream basal ganglia circuits, and then motor dysfunction with resting tremor, tonus, postural instability, and bradykinesia (Tolosa et al., 2006; De Virgilio et al., 2016). The pathogenesis of PD is still not fully understood, and genetic, environmental, and age factors are currently recognized as relevant to the development of PD (Emamzadeh and Surguchov, 2018). Although the link between PD and professions is mostly focused on environmental factors, some professions may increase the risk of PD due to exposure to environmental factors, such as toxins in agriculture or infections in the healthcare industry. However, there are still studies indicating that receiving a high level of education is a risk factor for Parkinson’s disease (Park et al., 2005). In addition, in one study, the use of highly complex data was associated with an increased risk of PD (Valdés et al., 2014). Despite various proposed mechanistic theories, the pathogenesis of PD remains unclear. Therefore, understanding the underlying pathogenesis of PD is essential to facilitate the development of effective treatments and improve prognosis.

In 1943, Cattell defined intelligence as composed of two factors: crystalline intelligence and fluid intelligence “(Horn and Cattell, 1967)”. Crystalline intelligence reflects the results of previous learning, while fluid intelligence is the ability to translate and solve problems. Fluid intelligence is considered independent of learning, experience, and education (Ghisletta et al., 2012). Research has found that fluid intelligence tends to decrease with age (Ghisletta et al., 2012) and this ability is impaired in dementia patients. However, there is little direct assessment of the causal effects of intelligence and fluid intelligence scores on PD. A recent cohort study based on a large population found that individuals with higher intelligence and higher education levels are more likely to develop Parkinson’s disease (Fardell et al., 2020). The connection between higher intelligence and PD may be due to higher fluid intelligence scores. However, as fluid intelligence scores are a powerful predictor of the combination of intelligence and education, intelligence may be a partial reason for the connection between fluid intelligence scores and PD. A previous study investigated the causal relationship between intelligence and several diseases in the European population, but did not find any statistical evidence that had an impact on PD (Shi et al., 2022). Therefore, we further explore the causal relationship between intelligence and fluid intelligence scores and PD through MR analysis.

Mendelian randomization (MR) is a method that has gained popularity in the medical research field in recent years (Emdin et al., 2017). It allows for estimating the causal impact of a modifiable risk factor using genetic variants from observational data. This method is favored due to its unique advantages and the rapid development of genomics. MR analysis provides a solution to the limitations of conventional studies by using single nucleotide polymorphisms (SNPs) as instrumental variables (IVs) to establish causality between a risk factor (exposure) and an outcome (Emdin et al., 2017). The MR method depends on three assumptions: (1) that the genetic variant is linked with the exposure of interest; (2) that the genetic variant is not affected by confounding factors; and (3) that the genetic variant indicators influence the outcome solely through the exposure (Skrivankova et al., 2021). The IVs utilized in MR analysis are derived via genome-wide association studies (GWAS), which are now available due to advancements in high-throughput genomic technologies. Therefore, in this study, we examined the causal effects of intelligence and fluid intelligence score on PD by using both MR and conventional analyses. This approach can provide estimates of the effects of the trait while reducing the bias due to confounding and reverse causation.

Therefore, to further understand the pathogenesis of PD, this study used public database data from IEU OpenGWAS database and the MR analysis method to explore the causal relationship between intelligence and fluid intelligence score and PD. This is crucial for an in-depth study of the basic mechanisms of PD, providing a new possibility for the treatment and improvement of the quality of life for PD.

## 2 Materials and methods

### 2.1 Data source

Three datasets, intelligence (ebi-a-GCST006250), fluid intelligence score (ukb-b-5238), and PD (ieu-b-7), were downloaded from the OpenGWAS database^1^ for the MR analysis in this study. The intelligence dataset was comprised of 33,674 cases and 449,056 controls, with 17,891,936 single nucleotide polymorphisms (SNPs). The intelligence dataset contained 269,867 samples and 9,276,181 SNPs, and the fluid intelligence dataset consisted of 149,051 samples and 9,851,867 SNPs.

### 2.2 Data preprocessing

In this study, SNPs with genome-wide significance (*p* < 5 × 10^–8^) were selected as instrumental variables (IVs) that were significantly associated with exposure factors. In addition, the IVs for linkage disequilibrium (LD) were removed in order to obtain genetically independent IVs (*R*^2^ = 0.001, kb = 10,000) (Zhao et al., 2022). The filtering process for IVs was performed via the extract_instruments function in TwoSampleMR package (Li et al., 2023).

### 2.3 MR analysis

In this study, the harmonise_data function in TwoSampleMR R package was used to harmonise effect alleles and effect size. MR analysis of causality was carried out by three main methods [MR Egger (Bowden et al., 2015), weighted median (Bowden et al., 2016), inverse variance weighted (IVW) (Burgess et al., 2015)], of which the IVW method was decisive and the most dominant. A causal relationship between exposure factors and outcome was indicated only when the *p*-value of the IVW method was less than 0.05. When assuming that all SNPs are valid IVs, the IVW method can provide a valid estimate of the causal relationship between exposure factors and outcome (Zhu et al., 2023). In addition, the results of MR analysis were visualized through scatter plots, forest plots and funnel plots.

### 2.4 Sensitivity analysis

The reliability of MR analysis was evaluated by sensitivity analysis, including heterogeneity tests, horizontal pleiotropy test and leave-one-out test (LOO). In the heterogeneity test, the Q-*p*-value greater than 0.05 indicated that there was no heterogeneity, and if there was heterogeneity, it was necessary to use the IVW test for random effects (Luo et al., 2023). Horizontal pleiotropy test was used to assess the presence of confounders in the MR analysis, with a *p*-value of the result greater than 0.05 indicating that there was no horizontal pleiotropy effect (Xu et al., 2022). To confirm the stability of the MR analysis, LOO analysis was carried out to find out if a single SNP could change the overall effects of all the SNPs (Cui et al., 2021).

### 2.5 Functional enrichment analysis of genes corresponding to SNPs

In order to explore the functions and pathways involved in the genes regulated by the IVs, the genes corresponding to the SNPs were first identified by the Variation effect predictor (VEP) of Ensembl website. The genes corresponding to SNP were included to enrichment analysis based on gene ontology (GO) and Kyoto Encyclopedia of Genes and Genomes (KEGG) via the clusterProfiler package in R and the screening condition was *p*-value < 0.05.

## 3 Results

### 3.1 Intelligence had a significant causal relationship with PD

**FIGURE 1:**
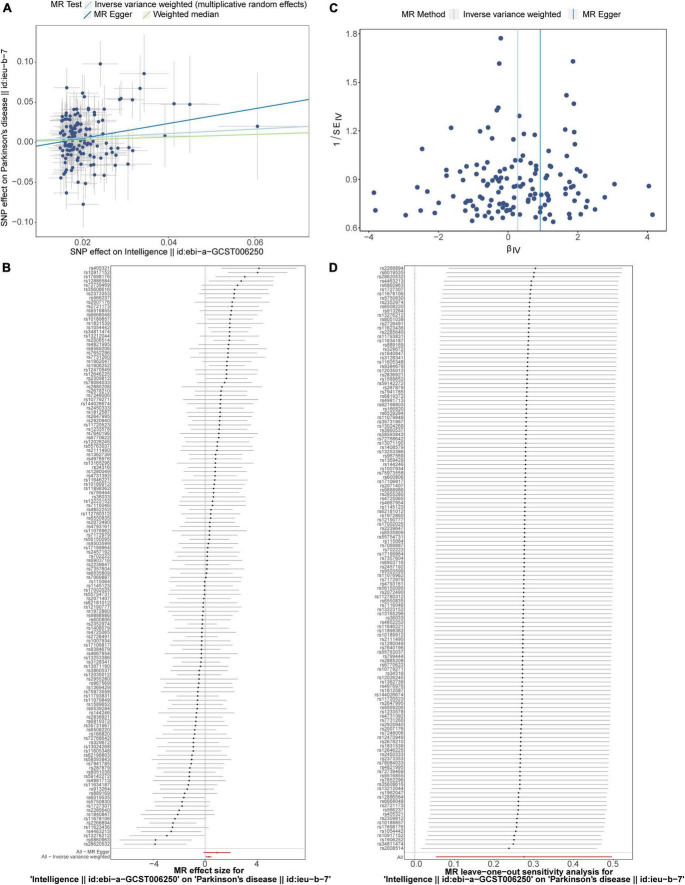
The MR estimates of the causal relationship between intelligence and PD. **(A)** The scatter plot of the association of intelligence on PD. **(B)** The forest plot of causal relationship between intelligence on PD. **(C)** The funnel plot of MR study between intelligence and PD. **(D)** LOO analysis of the association of intelligence on PD. MR, Mendelian randomization; PD, Parkinson’s disease; SNP, single-nucleotide polymorphism; LOO, leave-one-out.

In this study, a total of 137 intelligence-related SNPs were screened out for MR analysis (Supplementary Table 1). IVW results indicated that there was a significant causal relationship between intelligence and PD (*p* = 0.015), and elevated intelligence indicators would increase the risk of PD [Odd Ratio (OR) = 1.316] (Table 1). In the scatter plot, the effect of SNPs of intelligence on PD was positively correlated overall, indicating that the risk of PD increases with the increase of intelligence indicators (Figure 1A). In the forest map illustrated that the effect values of exposure factors on outcome variables were all greater than 0, indicating that the increase of intelligence index would increase the risk of PD, and intelligence was the cause and PD was the effect (Figure 1B). The funnel plot illustrated that MR analysis of intelligence and PD were consistent with Mendel’s second random law (Figure 1C).

**TABLE 1:** MR analysis of intelligence and Parkinson’s disease.

id.exposure	id.outcome	Outcome	Exposure	Method	nsnp	b	se	*p*-value	lo_ci	up_ci	or	or_lci95	or_uci95
ebi-a-GCST006250	ieu-b-7	Parkinson’s disease || id:ieu-b-7	Intelligence || id:ebi-a-GCST006250	MR Egger	137	0.921215	0.53027	0.084622	−0.11811	1.960544	2.512342	0.888595	7.10319
ebi-a-GCST006250	ieu-b-7	Parkinson’s disease || id:ieu-b-7	Intelligence || id:ebi-a-GCST006250	Weighted median	137	0.164931	0.140141	0.239237	−0.10974	0.439607	1.179312	0.896063	1.552097
ebi-a-GCST006250	ieu-b-7	Parkinson’s disease || id:ieu-b-7	Intelligence || id:ebi-a-GCST006250	Inverse variance weighted (multiplicative random effects)	137	0.274955	0.113018	0.014981	0.05344	0.496471	1.316472	1.054893	1.642914

The reliability of the results of the MR analysis of intelligence and PD was then assessed through sensitivity analysis. The quantitative results of the Cochran’s Q test for the IVW method indicated that there was heterogeneity (Q-*p*-value = 4.435 × 10^–4^) (Table 2). The *p*-value of the MR-Egger test was 0.214 in horizontal pleiotropy test, indicating that there were no confounding factors in this MR analysis, which further ensured the reliability of the MR results (Table 3). Finally, in the forest map, each SNP was gradually eliminated through LOO analysis, and the effect of the remaining SNPs on the outcome variable did not change much, indicating that the results of the MR analysis were reliable and stable (Figure 1D). Overall, the above results showed that there was a significant causal relationship between intelligence and PD and that intelligence was a risk factor for PD.

**TABLE 2:** Heterogeneity test for intelligence-Parkinson’s disease.

id.exposure	id.outcome	Outcome	Exposure	Method	Q	Q_df	Q_*p*-value
ebi-a-GCST006250	ieu-b-7	Parkinson’s disease || id:ieu-b-7	Intelligence || id:ebi-a-GCST006250	MR Egger	195.3482	135	0.00053
ebi-a-GCST006250	ieu-b-7	Parkinson’s disease || id:ieu-b-7	Intelligence || id:ebi-a-GCST006250	Inverse variance weighted	197.5993	136	0.000443

**TABLE 3:** Tests of Multiple Validity of Intelligence-Parkinson’s disease Levels.

id.exposure	id.outcome	Outcome	Exposure	egger_intercept	se	*p*-value
ebi-a-GCST006250	ieu-b-7	Parkinson’s disease || id:ieu-b-7	Intelligence || id:ebi-a-GCST006250	−0.01331	0.010674	0.214455

### 3.2 Genes corresponding to intelligence-related to SNPs were mainly enriched in synaptic vesicle cycle and polycomb repressive complex, etc.

**FIGURE 2:**
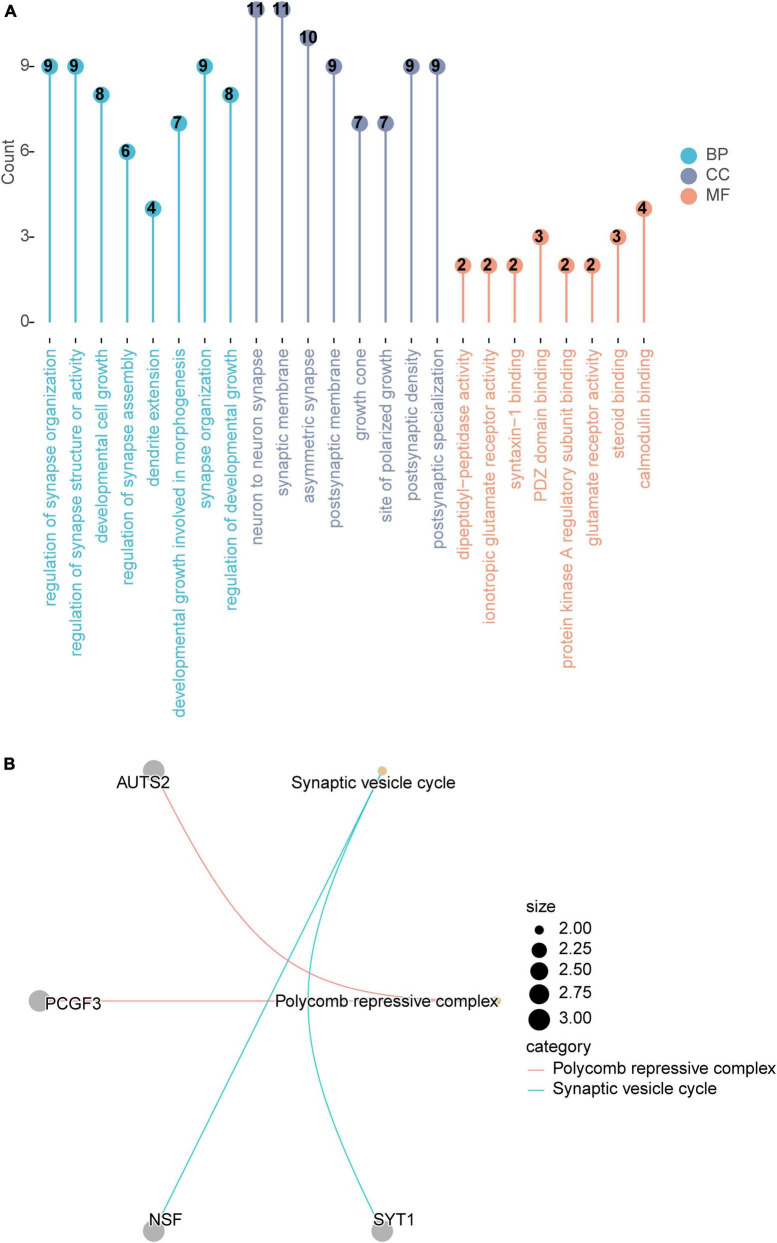
Functional enrichment of genes corresponding to SNPs. **(A)** GO enrichment analysis. **(B)** KEGG enrichment analysis. PD, Parkinson’s disease; GO, gene ontology; KEGG, Kyoto Encyclopedia of Genes and Genomes.

In this study, a total of 87 genes corresponding to intelligence-related SNPs were identified (Supplementary Table 2). Based on the GO database, these genes were enriched in a total of 343 entries, of which 241 entries were significantly enriched for biological processes (BP), 60 for cellular components (CC), and 42 for molecular functions (MF) (Supplementary Table 3). Among them, the most significant enrichment was found in relevant entries such as regulation of synapse organization, developmental cell growth and so on (Figure 2A). Moreover, a total of two signaling pathways were significantly included in KEGG enrichment analysis for these genes, namely synaptic vesicle cycle and polycomb repressive complex (Figure 2B).

### 3.3 There was a significant causal relationship between fluid intelligence score and PD

**FIGURE 3:**
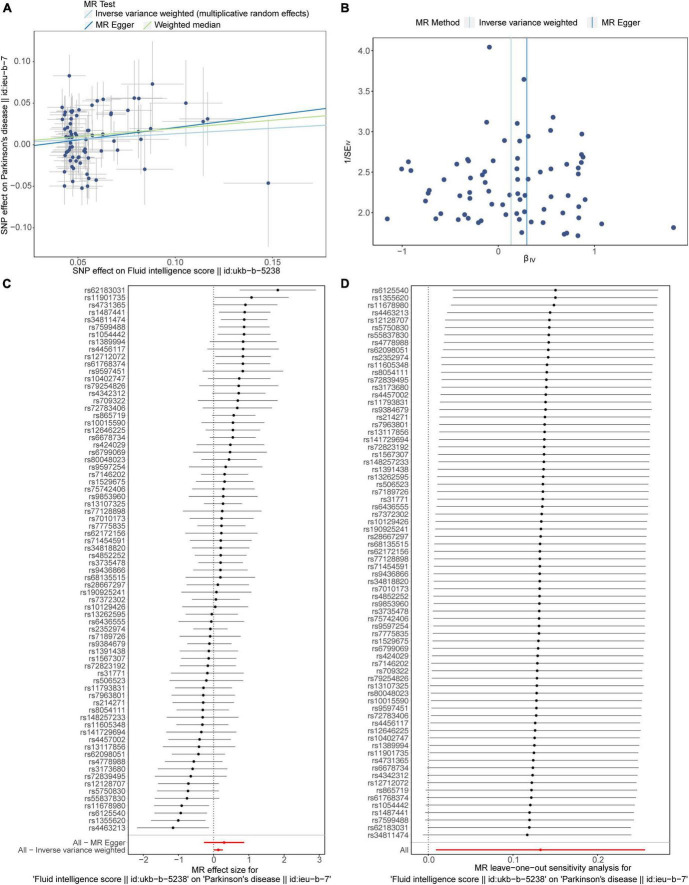
The MR estimates of the causal relationship between fluid intelligence scores and PD. **(A)** The scatter plot of the association of fluid intelligence scores on PD. **(B)** The forest plot of causal relationship between intelligence scores on PD. **(C)** The funnel plot of MR study between intelligence scores and PD. **(D)** LOO analysis of the association of fluid intelligence scores on PD. MR, Mendelian randomization; PD, Parkinson’s disease; SNP, single-nucleotide polymorphism; LOO, leave-one-out.

First, there were 74 SNPs related to fluid intelligence score were screened out for MR analysis of fluid intelligence score and PD (Supplementary Table 4). In MR analysis, the *p*-value of IVW algorithm was 0.035 and the OR value was 1.142, indicating that fluid intelligence score was causally related to PD, and fluid intelligence score was a risk factor (Table 4). The slope of the IVW line in the scatter plot was positive and the intercept was close to 0, indicating that the risk of PD was promoted with the increase of fluid intelligence score (Figure 3A). The same result was demonstrated in the forest plot, where the overall effect value of the fluid intelligence score on the PD variables was greater than 0 (Figure 3B). Finally, the funnel plot demonstrated that the Mendelian analysis of fluid intelligence score and PD was in accordance with the Mendelian law of randomness (Figure 3C).

**TABLE 4:** MR analysis of fluid intelligence scores and Parkinson’s disease.

id.exposure	id.outcome	Outcome	Exposure	Method	nsnp	b	se	*p*-value	lo_ci	up_ci	or	or_lci95	or_uci95
ukb-b-5238	ieu-b-7	Parkinson’s disease || id:ieu-b-7	Fluid intelligence score || id:ukb-b-5238	MR Egger	74	0.297327	0.290427	0.309376	−0.27191	0.866564	1.346256	0.761923	2.378724
ukb-b-5238	ieu-b-7	Parkinson’s disease || id:ieu-b-7	Fluid intelligence score || id:ukb-b-5238	Weighted median	74	0.196875	0.079343	0.01309	0.041363	0.352388	1.217592	1.04223	1.42246
ukb-b-5238	ieu-b-7	Parkinson’s disease || id:ieu-b-7	Fluid intelligence score || id:ukb-b-5238	Inverse variance weighted (multiplicative random effects)	74	0.132545	0.062966	0.035288	0.009132	0.255958	1.141731	1.009174	1.291699

In the sensitivity analysis, the Q-*p*-value for IVW by Cochran’s Q quantification was less than 0.05 (Q-*p*-value = 2.609 × 10^–4^), indicating the presence of heterogeneity (Table 5). The *p*-value of horizontal pleiotropy test was 0.563, indicating that there was no horizontal pleiotropy (Table 6). Finally, the reliability of the MR analysis of fluid intelligence scores with PD was demonstrated by the LOO method, which illustrated that the results did not change much after the exclusion of each SNP (Figure 3D). These analyses indicated that there was a causal relationship between fluid intelligence scores and PD, and high fluid intelligence score was associated with an increased risk of PD disease.

**TABLE 5:** Heterogeneity test for fluid intelligence score-Parkinson’s disease.

id.exposure	id.outcome	Outcome	Exposure	Method	Q	Q_df	Q_*p*-value
ukb-b-5238	ieu-b-7	Parkinson’s disease || id:ieu-b-7	Fluid intelligence score || id:ukb-b-5238	MR Egger	121.8352	72	0.000223
ukb-b-5238	ieu-b-7	Parkinson’s disease || id:ieu-b-7	Fluid intelligence score || id:ukb-b-5238	Inverse variance weighted	122.4071	73	0.000261

**TABLE 6:** Multiple validity test for fluid intelligence score-Parkinson’s disease level.

id.exposure	id.outcome	Outcome	Exposure	egger_intercept	se	*p*-value
ukb-b-5238	ieu-b-7	Parkinson’s disease || id:ieu-b-7	Fluid intelligence score || id:ukb-b-5238	−0.00908	0.015615	0.56283

### 3.4 Genes corresponding to fluid intelligence score-related to SNPs played important roles in developmental growth and Alzheimer’s disease, etc.

**FIGURE 4:**
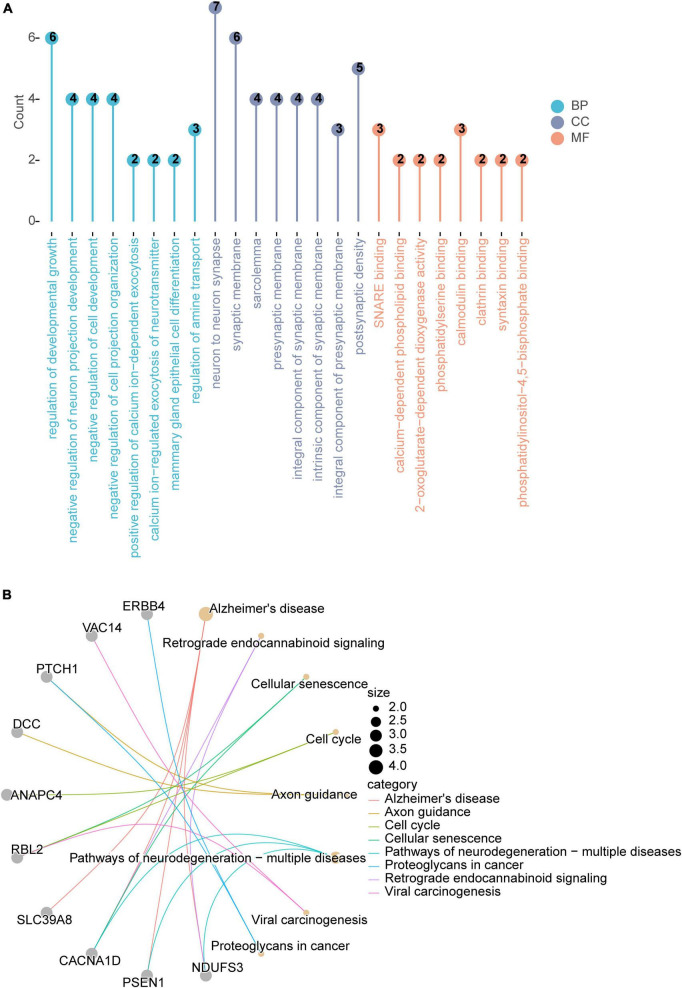
Functional enrichment of genes corresponding to SNPs. **(A)** GO enrichment analysis. **(B)** KEGG enrichment analysis. PD, Parkinson’s disease; GO, gene ontology; KEGG, Kyoto Encyclopedia of Genes and Genomes.

Functional enrichment analysis was performed on 44 genes corresponding to 74 fluid intelligence score related to SNPs (Supplementary Table 5). Firstly, a total of 454 items were enriched in GO enrichment analysis, among which 299 items were significantly enriched in BP, 67 items were significantly enriched in CC and 39 items were significantly enriched in MF (Supplementary Table 6). The main enriched entries of these genes included regulation of developmental growth, negative regulation of neuron projection development, etc (Figure 4A). In addition, these genes are mainly enriched in KEGG to 8 signaling pathways such as Alzheimer’s disease, cellular senescence and so on (Figure 4B).

## 4 Discussion

With the improvement of medical level and technological progress, the average life expectancy of humans is increasingly extended. Therefore, the so-called “aging-related disorders” (such as related cardiovascular diseases, central nervous system degenerative diseases, and malignant diseases) have gradually attracted the attention of the medical community (Roth et al., 2018; Janić et al., 2019). Neurodegenerative diseases have become an important research topic for scientists. PD is a neurodegenerative disease characterized by decreased motor function, but the etiology of PD remains largely unknown, making it considered an incurable chronic neurological disease. However, researchers are still committed to seeking potential disease improvement treatments. Many studies have attempted to demonstrate the relationship between cognition, intellectual development, and PD. Establishing associations using clinical trial design is indeed prone to various biases due to limited sample size and ethical and financial challenges. In order to strengthen causal inference, we conducted an MR study. In this study, based on the large-scale GWAS data from Europe, we evaluated the possible causal relationship between intelligence, fluid intelligence scores and the risk of developing PD using multiple MR methods. According to our research, higher intelligence and fluid intelligence scores were positively correlated with the risk of developing PD in the European population.

In general, low levels of education, intelligence, and socio-economic status are closely related to Alzheimer’s disease risk. According to the hypothesis that high intelligence affects health outcomes through the excellent ability to prevent and manage diseases, individuals with higher scores of intelligence and fluid intelligence should be at lower risk of illness (Fardell et al., 2020). However, in PD, we obtained the opposite situation. Our research results are consistent with previous case-control studies (Fardell et al., 2020). So we need to determine the link between high intelligence and the risk of PD, although there is currently little knowledge of the underlying mechanisms, the link between intelligence and PD is reasonable. Firstly, research has shown that intelligence is not only determined by genetic factors, but also by environmental factors such as infections, toxins, trauma, etc. All of these environmental factors are considered closely related to the etiology of PD (Wirdefeldt et al., 2011). Secondly, the relationship between higher intelligence and higher risk of PD may be related to lifestyle. Studies have shown that individuals with higher intelligence or education levels have lower cholesterol levels (Lara and Amigo, 2018), which is associated with increased PD risk (Huang et al., 2015), People with high intelligence and fluid intelligence may be able to resist high cholesterol intake through learning. Smoking cessation is a known protective factor for PD (Breckenridge et al., 2016; Delamarre and Meissner, 2017), and individuals with high intelligence and fluid intelligence are less likely to smoke or have increased resistance to smoking. Adults with lower levels of education are more likely to smoke due to psychological factors such as inferiority and stress, or due to tobacco advertising. The main reason for their difficulty in quitting smoking may be their lack of motivation, social support, and sufficient resources to purchase nicotine replacement products (Hiscock et al., 2012). The mediating role of these factors in the relationship between intelligence (IQ) and PD cannot be completely denied, and further research is needed.

Secondly, people with higher scores in intelligence and fluid intelligence are mostly engaged in advanced management, teaching, or other complex tasks with high difficulty and pressure. Studies have shown that people who engage in more complex tasks such as data analysis and processing, especially men, are at higher risk of developing PD (Zhang and Lang, 2023). Jobs involving higher occupational complexity, such as senior management and professor roles, require more mental activity and may also involve higher levels of stress (Wood et al., 2004). Work pressure can lead to an increase in glutamate levels (Wood et al., 2004), which is related to PD (Mitchell et al., 1989). Some cognitive symptoms of PD may be related to primary neurodegeneration in the cortex and higher cognitive regions, or may be mediated by abnormal neural activity (McGregor and Nelson, 2019). So, a person’s intelligence and fluid intelligence scores may affect their profession, lifestyle, income, living environment, etc. Anyway, as intelligence is a complex quantification method and may be influenced by other factors not considered or discussed in this study, future research is needed to validate this finding. If confirmed, further efforts are needed to investigate the potential mechanisms linking intelligence and PD.

Another prominent research direction for PD is to evaluate genetic changes, including single gene mutations and single nucleotide polymorphisms (SNPs). Many single gene mutations have been identified that lead to or increase the risk of PD, including coding α- Genes of synaptic nucleoprotein, leucine rich repeat kinase 2 (LRRK2) (Bardai et al., 2018), glucose cerebrosidase, parkin, and PTEN induced putative kinase 1 (PINK-1) (Yang et al., 2022). The Whole Genome GWAS has also identified many SNPs that increase the risk of PD, which have been combined with smaller disease association studies to form a meta-analysis database (Nalls et al., 2019). To explore the potential relationship between intelligence and fluid intelligence score related genes associated with SNP and SNP in PD, we analyzed multiple genes and signaling pathways through GO and KEGG analysis, among which synaptic vesicle function is particularly important. Synaptic dysfunction has been identified as an early neuropathological event in PD. Synapses mainly rely on adhesion between neurons, glial cells, and extracellular matrix (Cingolani et al., 2008). The pathway analysis of PD has identified frequent SNPs in cell adhesion pathways, indicating that cell adhesion dysfunction may play a role in disease pathology (Nalls et al., 2019). And our study also obtained similar results. Synapse loss is strongly associated with cognitive decline in human and animal models of AD (Tzioras et al., 2023). Studies have shown that homeostatic control of synaptic connections plays a central role in the etiology and treatment of depression (Duman and Aghajanian, 2012). Through GO enrichment analysis, we found that synapses play an important role in intelligence and fluid intelligence scores. The human brain contains trillions of synapses, but synaptic density and plasticity decrease with age, leading to a slowing down of human responses, which has been demonstrated in AD mouse models (Aguado et al., 2024; Richardson et al., 2024). Which may be our future research direction, such as the development of new synaptic biomarkers to diagnose PD early.

This study was the first to use MR analysis to examine the causal relationship between intelligence, fluid intelligence scores and PD. However, this study still had certain limitations. Firstly, this study cannot completely exclude other potential factors, such as occupation and family income, family social status, and lifestyle. Therefore, more MR tools were needed to rule out the potential role of these factors in the causal relationship between intelligence level, fluid intelligence score, and PD. Secondly, this study only covered the European population and still requires updated MR analysis based on large-scale GWAS aggregated data from other races and more genetic tools to validate.

## 5 Conclusion

In this study, we demonstrated by MR analysis that increased intelligence increases the risk of developing PD, while fluid intelligence score is an important risk factor for the development of PD, providing important genetic support for the causal relationship between PD and intelligence, fluid intelligence score. In addition, gene enrichment analysis revealed that synaptic vesicles had an important role between the two, providing new references and evidence for the study and treatment of PD.

## Data availability statement

Raw data for the MR analysis in this study can be found online in the OpenGWAS database1. Other data used in this study can be found in this article/Supplementary material. Further inquiries can be directed to the corresponding author/s.

## Author contributions

CJ: Conceptualization, Data curation, Formal analysis, Funding acquisition, Investigation, Methodology, Project administration, Resources, Software, Supervision, Validation, Visualization, Writing – original draft, Writing – review & editing. XZ: Conceptualization, Data curation, Formal analysis, Investigation, Resources, Supervision, Validation, Writing – original draft, Writing – review & editing. XM: Conceptualization, Investigation, Project administration, Supervision, Validation, Visualization, Writing – review & editing. HX: Formal analysis, Funding acquisition, Validation, Visualization, Writing – review & editing.

## Footnotes

1
https://gwas.mrcieu.ac.uk/
